# Maternofoetal Complications and Their Association with Proteinuria in a Tertiary Care Hospital of a Developing Country

**DOI:** 10.1155/2014/431837

**Published:** 2014-04-14

**Authors:** Archana Kumari, Avinash Chakrawarty, Abha Singh, Ritu Singh

**Affiliations:** ^1^Department of Obstetrics and Gynaecology, Lady Hardinge Medical College and Associated Hospitals, New Delhi 110001, India; ^2^Department of Medicine, All India Institute of Medical Sciences, New Delhi 110029, India; ^3^Department of Biochemistry, Lady Hardinge Medical college and Associated Hospitals, New Delhi 110001, India

## Abstract

*Objective*. To investigate association between maternofoetal complications and the amount of proteinuria measured by spot urine protein creatinine ratio in patients with preeclampsia. * Methods*. 200 consecutive patients with preeclampsia were recruited in the study. The complications like first episode of severe hypertension, renal insufficiency, raised level of aspartate transaminase, signs of neurological involvement, thrombocytopenia, eclampsia, and need to shift in intensive care units were studied. The maternal outcome was studied in terms of type of labour, outcome of pregnancy, mode of delivery, indication of cesarean section, and maternal mortality. The foetal complications and outcome parameters were birth weight, Apgar score at the time of birth and at five minutes, need of high dependency unit care, and perinatal mortality. * Result*. The frequency of various maternal and foetal complications was between 14–53% and 22–92%, respectively. Maternal mortality was 3%, whereas perinatal mortality was 23%. Statistically significant association was found between the frequencies of various complications in mother and newborn and spot UPCR. * Conclusion*. The rate of various maternofoetal complications in preeclampsia is higher in developing countries than in developed world. Maternofoetal complications and outcome correlate with maternal spot UPCR.

## 1. Introduction

Preeclampsia is a major health problem in maternal health with the prevalence ranging from 1.8% to 16.7% in developing countries [1]. This multisystem disorder affects millions of women worldwide and is recognized as an important direct cause of maternal and foetal morbidity and mortality [2].

The course, prognosis, and outcome of this disease are quite heterogeneous and there exists great difference in the morbidity and outcome in the patients of developing and developed world. While there is lack of data from developing countries, studies from the developed world have reported complications in less than 10% [3–5]. Recently, great interest has been shown by investigators all around the world to find out predictors/markers that can predict the maternofoetal outcome in patients with preeclampsia [6–8]. A couple of studies have also been done to find out association between the amount of proteinuria and maternofoetal outcome in patients with preeclampsia but the results have been variable [9–11].

At our centre, we have found relatively high prevalence of maternofoetal complications in patients with preeclampsia. This study was carried out in patients with preeclampsia to estimate the prevalence of maternal and foetal morbidity and to investigate the association between the amount of proteinuria measured by spot UPCR and maternofoetal outcome.

## 2. Materials and Methods

The study was approved by the Institutional Ethics Committee of Lady Hardinge Medical College on December 11, 2007, and written informed consent was taken in the approved format before enrolment of cases. This prospective observational study was done at the Department of Obstetrics and Gynaecology in collaboration with the Department of Biochemistry in Lady Hardinge Medical College and associated S.S.K. Hospital, New Delhi. 200 consecutive pregnant women with symptoms and signs suggestive of preeclampsia were enrolled from January 2008 to February 2010.

In the present study, preeclampsia was diagnosed in patients who had resting blood pressure equal to or more than 140/90 mmHg at two different occasions at least 4 hours apart with significant proteinuria detected in urine samples (defined as more than or equal to 300 mg/24 hrs or spot UPCR 0.3 gm/gm in random urine sample). We excluded patients with chronic hypertension, diabetes mellitus, preexisting renal disease, urinary tract infection, intrauterine foetal death, multiple-foetus gestations, premature rupture of membrane, and postterm pregnancy from our study. Postterm pregnancies were excluded from the study, as many of them needed urgent termination of pregnancy.

5–10 mL of voided midstream urine sample was collected for measurement of spot UPCR. Urine protein concentration was estimated on Synchron CX-9 automated analyser from Beckman, using the kits obtained from Randox. Urine creatinine concentration was measured by modified Jaffe's reaction which is based on the principle that creatinine in alkaline solution reacts with picric acid to form a coloured complex. The amount of colour formed is directly proportional to the creatinine concentration. The urine protein: creatinine ratio was calculated by dividing value of urine protein in gm/dL by urine creatinine in gm/dL.

During the hospital stay, each patient was managed as per the standard protocol of the Institute. Blood pressure was checked every four to six hours and close monitoring was done for ominous clinical findings such as headache, visual problems, epigastric pain, rapid weight gain, oliguria, and vomiting. Fetal weight and amniotic fluid volume were evaluated. Doppler USG or biophysical scoring was done whenever indicated. Fluid input/output charting was done. All women in the study were followed until delivery and maternal and foetal complications were noted.

The maternal complications were studied in terms of (i) new episode of severe hypertension (≥170/110 mmHg), (ii) renal insufficiency (serum creatinine > 1.2 mg/dL) or oliguria (<400 mg/d), (iii) increased liver enzyme (AST > 40 U/L), (iv) signs of neurological involvements (hyperreflexia with clonus), (v) presence of thrombocytopenia (platelet count < 150 × 10^9^/L), (vi) episode of eclampsia, and (vii) need to shift in ICU.

The maternal outcome was studied in terms of (i) type of labour—spontaneous or induced, (ii) outcome of pregnancy—full term live birth or preterm live birth or intrauterine death, (iii) mode of delivery—normal or caesarean, (iv) indication of lower segment caesarean section (LSCS), and (v) maternal mortality.

The foetal complications and outcome parameters were (i) birth weight, (ii) Apgar score at birth and at 5 minutes, (iii) need for shift in HDU, and (iv) perinatal mortality.

*Statistical Test.* Independent *t*-test and one-way analysis of variance were used to find out association between spot UPCR and different maternal and foetal complications in patients of preeclampsia. Multiple comparison tests were done by using bonferroni corrections. A *P* value of <0.05 is considered to be statistically significant. Box and whisker plots were made to see the significant association between proteinuria and various complications.

## 3. Results

200 consecutive patients of preeclampsia were enrolled in the study. The general characteristics of the patients are mentioned in Table 1. The mean age of the patients was 24.3 years. More than two-thirds of the patients were multigravida. Almost one–third of the patients had severe hypertension on admission.

Table 2 shows the frequency of various maternal complications in the patients with preeclampsia and their relationship with the amount of proteinuria. Of the 200 hundred patients of preeclampsia, 44% developed severe hypertension, 21% developed raised liver enzymes, and 14% developed renal insufficiency, whereas 23% of patients developed thrombocytopenia. 53% of the patients developed signs of neurological involvement (like hyperreflexia or clonus) and 31% of the patients developed eclampsia. The maternal mortality in our study was 3%. The mean value of proteinuria was more statistically significant among the patients with complications like severe hypertension, raised liver enzymes, eclampsia, and need to shift in ICU. However, renal insufficiency, signs of neurological symptoms, thrombocytopenia, and maternal mortality showed no relationship with the amount of proteinuria.

Table 3 shows the outcome of labour in patients of preeclampsia and its association with the amount of proteinuria. 21% of the cases underwent spontaneous labour whereas in 79% induction of labour was done. 63% of the total patients delivered vaginally while 37% delivered by caesarean section. Of all the caesarean sections, more than 90% were emergency caesarean sections. A statistically significant association was found between the incidence of induced labour and amount of proteinuria present (*P* value = 0.02). Caesarean section was also associated with high amount of proteinuria (*P* value = 0.01).

Table 4 shows the frequency of the various foetal complications and outcome in patients of preeclampsia and its association with the amount of proteinuria. Out of 200 deliveries, 26 (13%) were still birth and 20 (10%) were early neonatal death. Of the rest, 74% of newborns were born with low birth weight; two-thirds of them were premature. 58% of newborns had to shift to neonatal HDU after birth. The perinatal mortality in our study was 23%. Low birth weight (prematurity), need to shift in neonatal HDU, and perinatal mortality rate were found to be statistically significantly associated with high amount of proteinuria in mother.

**Figure 1:**
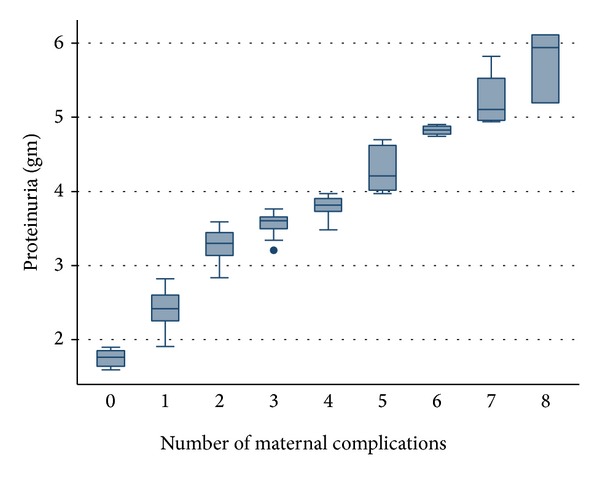
The relationship between the numbers of maternal complications and amount of proteinuria (measured as spot UPCR as gm/gm) in the patients of preeclampsia.

Figure 1 shows the relationship between the numbers of maternal complications and amount of proteinuria (measured as spot UPCR as gm/gm) in the patients of preeclampsia. The diagram shows that progressive increase in amount of proteinuria is associated with increase in the number of complications.

**Figure 2:**
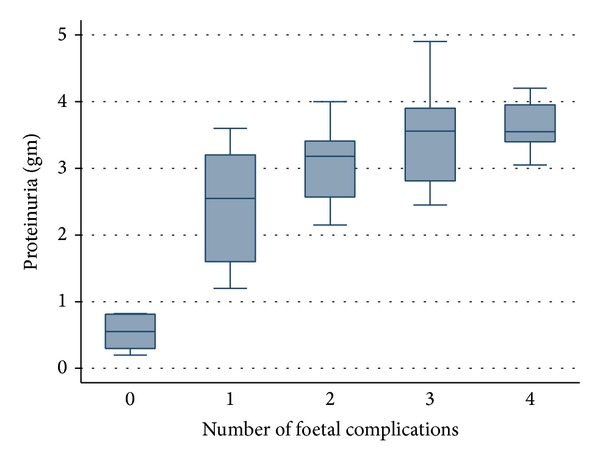
The relationship between the numbers of fetal complications and amount of proteinuria (measured as spot UPCR as gm/gm) in the patients of preeclampsia.

Figure 2 shows the relationship between the numbers of fetal complications and amount of proteinuria (measured as spot UPCR as gm/gm) in the patients of preeclampsia. The diagram shows that progressive increase in amount of proteinuria is associated with increase in the number of complications.

## 4. Discussions

There has been considerable interest among researchers to find out the markers that can predict maternofoetal complications or outcome of labour in patients with preeclampsia. Several factors and candidate biomarkers have been studied that include blood cells, blood pressure measurement, maternal age, serum uric acid, and amount of protein and proteomics in urine [6–8, 12, 13]. The results have been variable in most of the studies. However, proteinuria estimation seems to be promising as a candidate biomarker in predicting maternal and foetal outcome in women with preeclampsia. In a nested case controlled study involving 946 patients from United Kingdom, women with preeclampsia and proteinuria (300–499 mg/24 h) were found to have higher complication rates than women with gestational hypertension and chronic hypertension without proteinuria. Adverse perinatal outcomes were also higher in the former group [14]. In another retrospective analysis of 670 patients done at a tertiary referral centre of Sydney (Australia), maternal and perinatal outcomes in proteinuric preeclampsia were compared with nonproteinuric hypertensive disease. In this, the proteinuric cohort had higher blood pressure, more frequent need of magnesium sulphate administration in the management, and more preterm and operative delivery. However, the perinatal mortality rate was lower in the babies of women with proteinuric preeclampsia in comparison with that of nonproteinuric women [15]. Yet in another systemic review of sixteen studies that involved 6749 women with preeclampsia it was found that amount of proteinuria is a poor predictor of either maternal or fetal complications [16]. In a study from UK, proteomics in urine were studied in 113 patients with preeclampsia of gestation periods 12–16, 20, and 28 weeks. Among these biomarkers like collagen alpha chain, fibrinogen alpha chain and uromodulin fragments were found to predict preeclampsia at gestational week 28 [17]. In another study proteomic profiling in urine samples from 284 women was analyzed and it was identified that specific fragments of serpina-1 and albumin have potential to be used as biomarkers of preeclampsia [18].

Although some of these studies suggest that patients with preeclampsia who had macroalbuminuria had poorer outcome, there is lack of consistency in predicting the different maternal and foetal outcome measures by measuring amount of proteinuria [10, 13–16, 19]. Besides, most of these studies established the qualitative association; none investigated the quantitative association or strength of association between the two. This study was designed to investigate whether the frequency of various maternal and foetal complications is associated with the amount of proteinuria.

Majority of our patients were relatively young (mean age 24.3 years) and multigravida (67.3%). The mean age of gestation at the time of presentation was 34.5 weeks. These findings are in accordance with the previous studies in which the mean age of gestation at the time of presentation was 34 weeks [19]. The prevalence of complications in our patients was high (up to 60%). There is a paucity of studies from developing world and the studies from the developed countries suggest that the maternal complications among patients of preeclampsia are less than 10% [3–5]. Frequencies of maternal complications like severe hypertension, raised liver enzymes, eclampsia, and need to shift in ICU were found to have statistical significant association with the amount of proteinuria in patients with preeclampsia. Though there is paucity of studies to investigate association between the individual complications, results of our study are in accordance with some of the previous studies in which proteinuria was associated with poor maternal outcome [10, 13–15, 20]. However, in some other studies measurement of proteinuria failed to predict the maternofoetal outcome [7, 11, 16].

In our study, the prevalence of caesarean section was 37%. We found statistically significant association between amount of proteinuria and caesarean section. Spontaneous labour was present in less than one-fourth of cases that underwent vaginal delivery. Majority of our patients had to undergo increased operative intervention, which was associated with the amount of proteinuria. Similar result was found in a couple of previous studies in which higher rate of caesarean section was observed [15, 19, 21].

The foetal outcome was studied in terms of birth weight, Apgar score, and need to shift in neonatal HDU. In our study, 74% of newborns were of low birth weight; of these 70% were premature. The proportion of low birth weight and prematurity had statistically significant association with amount of protein in urine. 58% of newborns had to be shifted to neonatal HDU after birth. The perinatal mortality in our study was 23%. These morbidity factors and perinatal mortality were significantly associated with maternal proteinuria. A couple of studies investigated association between maternal proteinuria and foetal outcome in patients of preeclampsia. In two of these studies, adverse foetal outcome was associated with amount of proteinuria [9, 19] whereas one study failed to establish any association between the two [21].

The limitation of our study was that it does not establish causal association, although it establishes significant association between amount of proteinuria and some of the maternofoetal morbidities.

Our study highlights two major findings. Firstly, the frequency of complications of preeclampsia in mother and foetus is significantly higher in our patients than what is mentioned in western literature. This can be because of other effect modifiers like nutritional deficiency, lack of awareness, late detection of complications and intervention, and so forth. Besides, there can be some genetic or racial factors that are not yet identified. Secondly, in our population, the frequencies of the maternofoetal morbidities were found to be associated with amount of proteinuria. Though there are a couple of studies that have similar findings, many of the studies including some of the systemic reviews have failed to find any association between maternofoetal outcome and proteinuria in patients of preeclampsia. The reason of the variation in the observation can be due to yet unidentified genetic or racial factors. Population based multicentric research is required in this part of the world to investigate this topic.

## 5. Conclusion

The prevalence of complications in mother and newborn was higher in our hospital based study. The maternofoetal complications and outcome of labour in preeclampsia were associated with maternal spot UPCR. Suitably designed cohort study is required to establish causal association.

## Figures and Tables

**Table 1:** General characteristics of the patients.

Serial number	Parameter	Value
1	Mean age in years (S.D.)	24.3 (2.6)
2	Primigravida : multigravida	0.56 : 1
3	Mean period of gestation in weeks (S.D.)	35.5 (2.7)
4	Diastolic blood pressure (at the time of admission)	
	90–99 mm of Hg	38%
	101–109 mm of Hg	32%
	DBP ≥ 110 mmHg	30%

**Table 2:** Frequency of maternal complications and spot UPCR in the patients of preeclampsia.

Serial number	Parameter	Present		Absent		*P* value
		Number	Proteinuria spot UPCR (gm/gm)	Number	Proteinuria spot UPCR (gm/gm)	
1	Severe hypertension	88 (44%)	3.48 ± 1.20	112	1.86 ± 0.90	0.001
2	Raised liver enzymes	42 (21%)	2.61 ± 1.70	158	2.12 ± 1.10	0.02
3	Renal insufficiency	28 (14%)	2.42 ± 1.93	172	3.01 ± 1.81	0.12
4	Signs of neurological involvement (hyperreflexia or conus)	106 (53%)	2.98 ± 1.42	94	2.76 ± 1.31	0.26
5	Thrombocytopenia	46 (23%)	3.08 ± 1.02	154	2.88 ± 0.94	0.22
6	Eclampsia	62 (31%)	3.92 ± 2.31	138	3.04 ± 1.62	0.002
7	Need to shift in ICU	76 (38%)	2.98 ± 1.53	124	2.46 ± 1.50	0.02
8	Maternal mortality	6 (3%)	2.69 ± 1.11	194	3.01 ± 3.01	0.45

**Table 3:** Maternal outcome and spot UPCR in the patients of preeclampsia.

	Parameters	Frequency	Proteinuria spot UPCR (gm/gm)	*P* value
1	Type of labour			
	Spontaneous	42 (21%)	2.88 ± 2.4	0.02
	Induced	158 (79%)	3.98 ± 2.75	

2	Mode of delivery			
	Vaginal	126 (63%)	2.84 ± 1.9	0.01
	Caesarean	74 (37%)	3.46 ± 1.24	

3	Outcome of labour			
	Full term live birth	86 (43%)	2.14 ± 1.05	0.001
	Preterm live birth	88 (44%)	2.69 ± 0.86^a^	
	Intrauterine death	26 (13%)	3.32 ± 1.12^b, c^	

4	Indication of caesarean section			
	Emergency caesarean section	68 (92%)	3.86	0.002
	Elective caesarean section	06 (8%)	3.38	

5	No Maternal mortality	194 (97%)	3.01 ± 3.01	0.45
	Maternal mortality	6 (3%)	2.69 ± 1.11	

^a^Comparison between full term live birth and preterm live birth (*P* Value: 0.001); ^b^comparison between preterm live birth and intrauterine death (*P* Value: 0.001); ^c^comparison between full term live birth and intrauterine death (*P* Value: 0.013). Overall outcome of labour is significantly associated with proteinuria (one way analysis of variance).

**Table 4:** Fetal complications and outcome and spot UPCR in the patients of preeclampsia.

Serial number	Parameters	Present		Absent		*P* value
		Number	Proteinuria spot UPCR (gm/gm)	Number	Proteinuria spot UPCR (gm/gm)	
1	Low birth weight	128 (71%)	3.42 ± 1.72	46	2.82 ± 1.51	0.04
	Small for gestation age	40 (22%)	3.08 ± 1.20	10	2.98 ± 0.89	0.81
	Prematurity	88 (49%)	3.58 ± 2.37	36	2.63 ± 1.41	0.03

2	Low Apgar score	166 (92%)	3.07 ± 0.59	08	2.64 ± 0.08	0.04
	At birth	166 (92%)	3.75 ± 1.25	08	2.43 ± 1.92	0.005
	At 5 minutes	116 (64%)	3.98 ± 1.83	58	2.65 ± 1.75	0.001

3	Need for shift in neonatal HDU	96 (53%)	3.21 ± 1.02	78	2.87 ± 0.98	0.03

4	Perinatal mortality	46 (23%)	3.80 ± 2.27	154	2.76 ± 1.73	0.001
	Still birth	26 (13%)	3.40 ± 1.23	20	3.02 ± 1.04	0.27
	Early neonatal death	20 (10%)	3.02 ± 1.19	26	3.40 ± 1.23	0.30
